# Pyrene-Based Co-Assembled Supramolecular Gel; Morphology Changes and Macroscale Mechanical Property

**DOI:** 10.3390/gels6020016

**Published:** 2020-05-15

**Authors:** Ka Young Kim, Mirae Ok, Jaehyeong Kim, Sung Ho Jung, Moo Lyong Seo, Jong Hwa Jung

**Affiliations:** 1 Department of Chemistry and Research Institute of Natural Sciences, Gyeongsang National University, Jinju 52828, Korea; rk5321@gnu.ac.kr (K.Y.K.); meilai97@naver.com (M.O.); rwgjjang7@gnu.ac.kr (J.K.); 2 Department of Liberal Arts, Gyeongnam National University of Science and Technology (GNTECH), Jinju 52725, Korea; sungho@gntech.ac.kr

**Keywords:** Supramolecular gel, Mechanical strength, Co-assembly

## Abstract

Two pyrene derivatives having the perylenediimide (**1**) or the alky chain (**2**) in the middle of molecules were synthesized. Co-assembled supramolecular gels were prepared at different molar ratios of 0.2, 0.5, and 0.8 equiv. of **2** to **1**. By SEM observation, the morphology of co-assembled supramolecular gels changed from spherical nanoparticles to three-dimensional network nanofibers as the ratio of **2** increased. In addition, the pyrene-excimer emission of co-assembled gels increased with increasing concentration of **2**, and was stronger when compared with the condition without **1** or **2**, indicating the formation of pyrene interaction between **1** and **2**. In addition, the sol-gel transition was found to be reversible over repeated measurement by tube inversion method. The rheological properties of co-assembled supramolecular gels were also improved by increasing the ratio of **2**, due to the increased nanoscale flexibility of supramolecular packing by introducing alkyl chain groups through heterogeneous pyrene interaction. These findings suggest that macroscale mechanical strength of co-assembled supramolecular gel was strongly influenced by nanoscale flexibility of the supramolecular packing.

## 1. Introduction

Supramolecular gels are composed of low-molecular-weight organic compounds and use self-assembly processes, and have thus been an area of research interest due to their potential applications in photonics [1,2,3], optoelectronics [4,5,6,7], photodynamic therapy [8,9,10], and sensors [11,12]. Although supramolecular gels can be obtained from polymers, preparation using a low-molecular-weight gelator offers several advantages, such as easy synthesis, reversibility, and better control over the resulting physical and chemical properties [13,14,15,16]. In general, supramolecular gels could be constructed through intermolecular non-covalent interactions, such as hydrogen bonding, π–π stacking, and hydrophobic interactions [12,17,18,19]. 

Recently, researchers have been investigating supramolecular gels composed of two or more molecule types, i.e., co-assembled supramolecular gels, to control the resulting physical properties [20,21]. As the two or more molecules co-assemble to form a three-dimensional network, their physical properties can be more easily controlled by varying the composition ratio of the components. In co-assembled supramolecular gels, the molecular arrangement of co-assembly system plays a vital role in optical, mechanical properties. Therefore, the design of building blocks that enable morph co-assembled gels is important for developing functional soft materials.

Fluorescent gels are also useful as soft materials that be made of small molecular gelators though the supramolecular assembly of a well-defined complex of molecules [22,23,24]. Hydrogels, in particular, can potentially be a useful three-dimensional scaffold for tissue engineering due to their flexibility and water retention ability, similar to biological tissues [25,26]. Such gels differ from conventional hydrogels or organogels in their light emitting properties, but retain their extended networks that can be extensively swollen with water or organic solvents. In addition, fluorescent gels that possess several unique features are potential applicable in fields of sensors [11,12], optical and electrical devices, imaging agents [22], in biomedicine or for memory and display devices [4,5,6,7].

The various small molecular fluorescent dyes such as perylenediimide (PDI) [27,28,29], tetraphenylethene (TPE) [30], thienoviologen [31], lanthanides transition metal ions [32,33,34], and quantum dots [35,36], are excellent emitters with intense fluorescence and small molecular sizes that facilitate conjugation to the bulk gel network. Among fluorescent moieties, pyrene and its derivatives have been extensively applied as probes for molecular aggregation in polymer solutions, micelles, liquid crystals, and supramolecular gels [37,38]. Pyrene, representative of π-conjugated moieties with high fluorescence and unique emission properties, have been deemed as suitable candidates for investigating molecular arrangements and co-assembled nanomaterials [39], due to their ability of excimer formation in both solution and solid state [40]. Thus, we report control of morphology and mechanical property of co-assembled supramolecular gels by varying the ratio of pyrene derivatives having a perylenediimide (**1**) or an alkyl chain group (**2**). 

## 2. Results and Discussion

**Figure 1:**
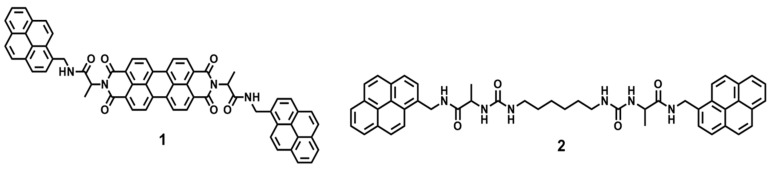
Chemical structures of compounds **1** and **2**.

Two pyrene derivatives having a perylenediimide (**1**) or an alkyl chain (**2**) were synthesized by a four- and one-step synthesis, respectively (Scheme S1 and Figure 1) The pyrene groups of compounds **1** and **2** were used as a linker to excimer formation in supramolecular gels. To increase flexibility in supramolecular gels, alkyl chain groups were introduced into compound **2**. These compounds were characterized by ^1^H NMR spectroscopy, high-resolution ESI mass spectroscopy, and IR spectroscopy (Figures S1–S5). 

**Figure 2:**
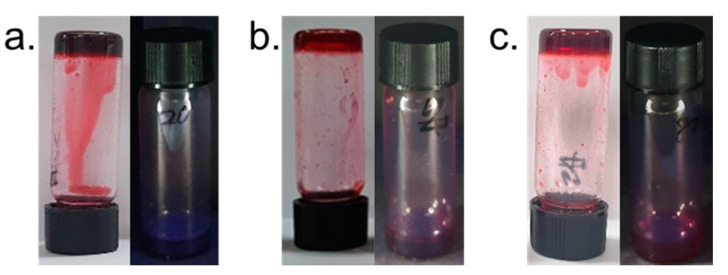
Photograph of co-assembled supramolecular gel dependent on the ratio of compound **2** in DMSO (1 wt %, with respect to **1**); [**2**] / [**1**] = (**a**) 0.2, (**b**) 0.5 and (**c**) 0.8.

The gelation ability of pyrene derivatives having a perylenediimide (**1**) at 0.5 equivalent of **2** was evaluated in various kinds of organic solvents. The gelation behavior was summarized in Table 1. A perylenediimide derivative **1** showed good solubility in chloroform resulting in no gel formation (Figure S6). On the other hand, formation of gel **1** in DMSO was confirmed at different equivalents of **2**. It was found out that compound **1** could be gelated DMSO at different molar ratios: (a) 1: 0.2, (b) 1: 0.5, and (c) 1: 0.8. This induced co-assembly by forming a pyrene-excimer between the pyrene groups, thus causing the transformation into a stable gel, as shown in Figure 2. The co-assembled supramolecular gels formed within 16 h, 30, and 80 min from the (a), (b), and (c) molar ratios, respectively. The gelation time was shortest when 0.5 equiv. of **2** was added to **1** solution, because the well-organized molecular arrangement to form excimer structure in co-assembled gel. In contrast, single-component solutions of either **1** or **2** did not form gel (Figure S7). These findings indicate that the formation of supramolecular gels was strongly dependent to the molar ratio between **1** and **2**. 

The tube inversion method with heating and cooling of co-assembled gel provided confirmation of reversible sol-gel transition. Co-assembled gel **1** seemed to break down from 119 °C at molar ratios [**2**]/[**1**] = 0.8. The sol-gel transition temperature of co-assembled gel in the presence of 0.8 equiv. of **2** was highest than those presence of 0.5 equiv. of **2** (Figure S8). Furthermore, the sol-gel transition was found to be reversible over repeated measurements. 

To demonstrate optical property, the co-assembled supramolecular gels of **1** + **2** in DMSO (1 wt%) were investigated by UV-vis and luminescence spectroscopy. The absorption spectrum of the co-assembled supramolecular gels centered at 345 nm, corresponding to the typical UV absorption band of pyrene (Figure S9) [41,42]. As the molecular ratio of **2** increased, no significant changes occurred in the absorption spectra, including that of the pyrene absorption. In contrast, the absorbance at 491 and 530 nm decreased with an increase in the concentration of **2**, those being the characteristic absorption bands of the PDI moiety of **1** [43,44]. 

**Figure 3:**
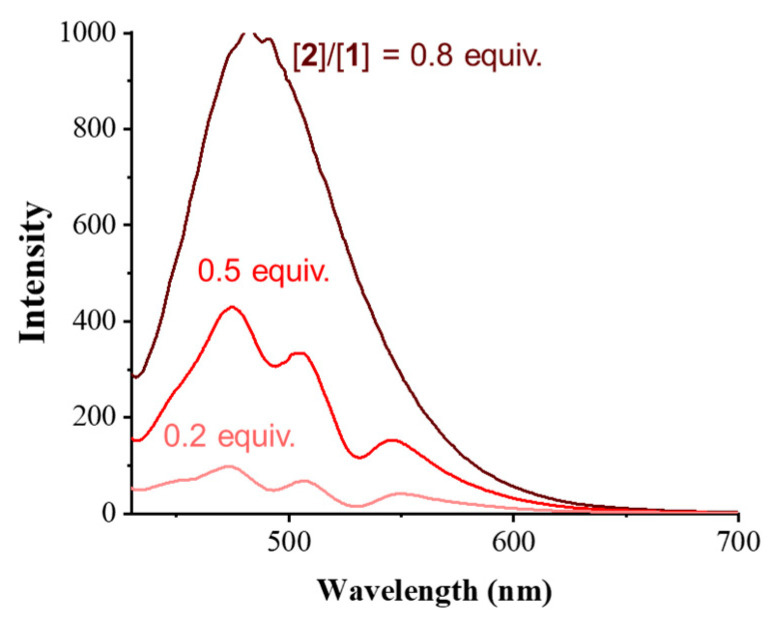
Luminescence spectra of co-assembled supramolecular gels (1 wt%) at a different molar ratio of **2** to **1** in dimethyl sulfoxide.

The luminescence spectra of the co-assembled supramolecular gels with excitation at 345 nm were also observed; the results are shown in Figure 3. As the molecular ratio of **2** increased, the luminescence intensity of co-assembled gels increased at 480 nm, indicating the formation of the pyrene-excimer. A weaker luminescence intensity change at the wavelength of the pyrene-excimer emission was observed in the luminescence spectra of the single-component solutions than in the co-assembled supramolecular gel under the same conditions, as shown in (Figure S10). These results suggest that a pyrene-excimer emission was formed with interaction between pyrene (**1**) and pyrene (**2**) in co-assembly process [45,46,47]. 

**Figure 4:**
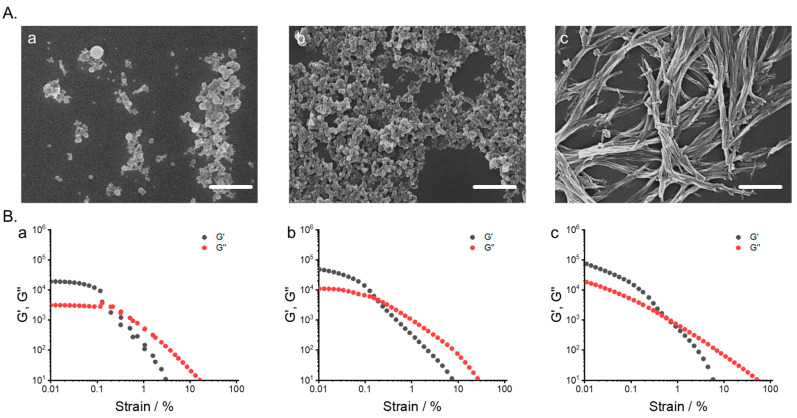
(**A**) SEM images of co-assembled supramolecular gel dependent on the ratio of compound 2 (scale bar, 1 μm). (**B**) Rheological properties (G’ black dot; G’’ red dot) of co-assembled supramolecular gel dependent on the ratio of compound 2. strain sweep tests at 0.001 % -100 %, [**2**]/[**1**]= (**a**) 0.2, (**b**) 0.5, and (**c**) 0.8 equivalents.

The morphologies of the co-assembled supramolecular gels were investigated using SEM and TEM; resulting images are shown in Figure 4A and Figure S11. The morphology of the co-assembled supramolecular gel at molar ratio = 0.2 exhibited a small spherical nanoparticle with a diameter of ca. 60 nm (Figure 4A (a)). At the middle 2:1 molar ratio studied, 0.5, spherical particles connected by a three-dimensional network were formed (Figure 4A (b) and Figure S11b). At 0.8 equiv. of 2, the resulting gel was a 3D network of nano-fibrous structures, as shown in Figure 4A (c) and Figure S11b. Distinct different morphologies were observed in the single-component solutions under the same conditions as supramolecular gels (Figures S12 and S13). Thus, an increase in the ratio of compound **2** would improve the flexibility of molecules in the supramolecular gel, thus leading to a 3D network of nanofiber forms of supramolecular nanostructures.

The rheological properties of the co-assembled supramolecular gels were then examined via rheometer measurements to determine the storage (G′) and loss (G″) moduli. Strain sweep, frequency sweep, and continuous step strain tests were conducted for three of the co-assembled supramolecular gels at 0.2, 0.5, and 0.8 equiv. of **2** (Figure 4B, Figures S14, S15 and S16). The results indicated that the moduli values gradually increased with an increasing concentration of **2**. G′ was greater than G″ when γ (G″:G′) was less than 0.1 % (Figure 4B). In contrast, when γ exceeded 0.1%, G′ decreased more than G″, indicating the breakdown of the gel network. This tendency suggested that the co-assembled supramolecular gels slid before breakdown. The performed frequency sweeps revealed a predominantly elastic character (G′ > G″) with almost constant G′ and G″ over the entire tested frequency range (0.6283–6283 rad s^−1^), demonstrating the quasi-solid property of the gels (Figures S14, S15, and S16A). A gel-to-quasi-liquid transition was reversible because G′ and G″ recovered quickly within 30 s when γ was reduced from 100% to 0.1%. When the amplitude oscillatory force was decreased at the same frequency (γ = 0.1% at 1.0 Hz), G′ rapidly increased, and the system returned to a quasi-solid state (tan δ = G″/G′ ≈ 0.26). Thus, the co-assembled supramolecular gel exhibited a rapid thixotropic response (Figure S16B). The recovery process involved the regeneration of the gel network. Significantly larger storage and loss moduli were observed in the resulting gel at higher molar ratios of **2**. These results suggest that introduction of nanoscale flexibility inside supramolecular gel enhanced macroscale mechanical strengths in co-assembly process.

## 3. Conclusions

In this work, we have demonstrated that morphology and mechanical property of co-assembled gels were dependent on the composition ratio of building blocks. The largest luminescence intensity of co-assembled gel was observed in a mixed solution when 0.8 equiv. of **2** was added to solution **1**, due to the well-organized molecular arrangement of excimer. The morphology of co-assembled supramolecular gels changed from spherical nanoparticles to three-dimensional network nanofibers as the ratio of **2** increased. In addition, the mechanical properties of co-assembled supramolecular gels was controllable by modification of the ratio of compound [**2**]:[**1**], due to the formation of pyrene-excimer and increased flexibility of supramolecular packing structure. Thus, further development of co-assembly supramolecular gels using the formation of pyrene-excimer can offer materials for optical applications and dual-functional gels by implementing functional derivatives.

## 4. Materials and Methods 

### 4.1. Reagents and Instruments

Unless otherwise noted, chemical reagents and solvents were purchased from commercial suppliers (Tokyo Chemical Industry, Tokyo, Japan, and Sigma-Aldrich, St. Louis, MO, USA), and used without further purification. The NMR spectra for ^1^H and ^13^C were taken on a Bruker DRX 300, and mass spectroscopy samples were observed using a JEOL (JMS-700, JEOL, Tokyo, Japan) mass spectrometer. A UV-visible spectrophotometer (Evolution 600, Thermo scientific, Waltham, MA, USA) was used to obtain the absorption spectra. IR spectra were observed over the range 500–4000 cm^−1^ using a Thermo scientific Nicolet iS10 infrared spectrometer. The fluorescence spectra were obtained using a RF-5301PC spectrophotometer (Shimadzu, Kyoto, Japan).

### 4.2. FE-SEM Observation

FE-SEM imaging was performed with a JEOL (JSM-7610F, JEOL, Tokyo, Japan) using an accelerating voltage 5 kV and an emission current of 8 μA. Samples were prepared by dropping dilute solutions of xero gel on a glass, then drying, and coating it with a thin layer of Pt to increase the contrast.

### 4.3. Rheological Properties

Rheological testing of the prepared gels was carried out using an AR-2000ex (TA Instruments Ltd, Newcastle, DE, USA) implemented with a 40-mm diameter parallel plate attached to a transducer. The gap in the setup for rheological testing of the gels was 1.0 mm and the experiments were conducted at 298 K. Strain sweep tests were performed with increasing amplitude oscillation up to 10% apparent strain on shear. Frequency sweeps were performed from 5 to 1000 Hz. The recovery properties of the gels in response to the applied shear force were investigated over 1500 s: 0.01% (300 s), then 1% (300–600 s), then 0.01% (600–900 s), then 1% (900–1200 s), and finally 0.01% (1200–1500 s).

### 4.4. Preparation of Co-Assembled Supramolecular Gel

Co-assembled supramolecular gels (16.54 mM, 1 wt%) were prepared at different molar ratio of 0.2, 0.5, and 0.8 equiv. of **2** to **1**. Compound **1** was placed in a 5 mL vial, where **2** (0.2, 0.5, and 0.8 equiv.) was added and then dissolved in DMSO (300 uL). The mixture was sonicated in a bath for a few seconds and heated until a clear solution was obtained. The samples were then maintained at room temperature to form the co-assembled supramolecular gel.

### 4.5. Synthesis of Compound 4

1-pyrenemethylamine (1.99 g, 8.590 mmol), 1-ethyl-3-(3-dimethylaminopropyl)carbodiimide (EDC, 3.23 g, 16.83mmol) and 1-hydroxybenzotriazole hydrate (HOBt, 1.52 g, 11.25 mmol) were added to a 100mL flask. Dichloromethane (30mL), DMF (10mL) and Triethylamine (4.70 mL, 33.67 mmol) were then injected into the flask. After the mixture was dissolved, *N*-(tert-Butoxycarbonyl)-L-Alanine (1.76 g, 9.3 mmol) in dichloromethane (10 mL) was added at 0 °C. The mixture was stirred for 1 h at 0 °C, and was heated up to room temperature. After stirring for 16 h room temperature, the mixture was extracted with dichloromethane and 10% HCl. The combined organics were washed with brine, dried (Na_2_SO_4_), and concentrated. The resulting crude material was purified by recrystallization from DCM/hexane to give a white solid (63.6 % yield). m.p. 180 °C; IR (KBr pellet): 3315, 3041, 2989, 2920, 2875, 1687, 1645, 1519, 1252, 1164, 845, 756cm^−1^, ^1^H NMR (300 MHz, DMSO) δ 8.51 (t, 1H), 8.38–8.04 (m, 9H), 7.00 (d, 1H), 5.02 (m, 2H), 4.05 (t, 1H), 1.38 (s, 9H), 1.23 (d, 3H). ^13^C NMR (75 MHz DMSO-d*_6_*) δ ppm 172.3, 157.2, 151.2, 140.8, 133.5, 129.7, 129.0, 128.5, 128.2, 126.8, 126.3, 125.6, 124.0, 123.7, 123,2, 122.5, 80.0, 54.5, 28.4, 17.5; ESI-MS (*m*/*z*): Calculated for C_25_H_26_N_2_O_3_ [M+H]^+^ 403.20, Found [M+H]^+^ 403.52.

### 4.6. Synthesis of Compound 3

To compound **4** (0.49 g, 1.225 mmol), 15 mL 35–37 % HCl in methanol (previously stirred until homogeneous) was added and stirred for 2 h. The solution was diluted with additional methanol, water, and then added NaOH until change color. The solution was stirred for 1h, and the resulting precipitate was filtered and washed with water. The filtered solid was dried under the vacuum condition to afford compound **3** as a white solid in 98% yield. m.p. 159 °C; IR (KBr pellet): 3272, 3033, 2962, 2926, 1636, 1541, 1522, 840, 711cm^−1^; ^1^H NMR (300 MHz, DMSO-d_6_) δ 8.50 (s, 1H), 8.42–8.04 (m, 9H), 5.04 (m, 2H), 3.35 (q, 1H), 1.89 (s, 2H), 1.19 (d, 3H); ^13^C NMR (75 MHz, DMSO) δ ppm 176.28, 133.58, 131.27, 130.79, 130.53, 128.52, 128.00, 127.87, 127.46, 126.97, 126.72, 125.70, 125.62, 125.20, 124.51, 124.42, 123.68, 50.95, 40.69, 22.30; ESI-MS (m/z): Calculated for C_20_H_18_N_2_O [M+H]^+^ 303.15, Found [M+H]^+^ 303.27.

### 4.7. Synthesis of Compound 1

Perylene-3,4,9,10-tetracarboxylic acid bisanhydride (0.3 g, 0.76 mmol), compound **3** (0.50 g, 1.64 mmol), zinc acetate (0.54 g, 2.96 mmol) and imidazole (12g, 1176mmol) were added to a 50 mL one-neck flask. The reaction mixture was stirred at 100 °C for 24 h. After cooling to room temperature, the mixture was extracted with CHCl_3_ and ^1^N HCl (twice). The solution was concentrated by evaporator. The solid was dried and the crude product was purified by column chromatography (CHCl_3_/acetone, 98:2 *v*/*v* %). Yield: 12%, IR (KBr pellet, cm^−1^): 3432, 2925, 2852, 1698, 1654, 1594, 1363, 1340, 1253, 547, 810, 749; ^1^H NMR (300 MHz, DMSO-d_6_) δ 8.53 (s, 2H), 8.33–7.91 (m, 26H), 5.62 (d, 2H), 5.02 (s, 4H), 1.80 (d, 6H); ^13^C NMR (75 MHz, CDCl_3_) δ ppm 168.48, 162.60, 132.13, 131.34, 131.07, 130.39, 129.48, 129.28, 128.01, 127.53, 127.05, 126.26, 125.63, 125.47, 124.83, 121.50, 119.91, 42.62, 29.72, 14.40; Element analysis: calculated for C_64_H_40_N_4_O_6_: C 80.0, H 4.2, N 5.8 Found: C 79.8, H 4.3, N 5.6. 

### 4.8. Synthesis of Compound 2

Compound **2** (10mg, 9.92 μmol) was dissolved in DMSO (276 μL) by heating. 1.6-Diisocyanato-heaxane (79.4 μL) was diluted in DMSO (120.6 μL). Next, 20 μL of the solution of 1.6-Diisocyanato-heaxane and Dibutyltin dilaurate (5.3 μL) were added to solution of compound **2**. The mixed solution was heated until it melted clearly. Finally, the sample was maintained at room temperature to form the supramolecular gel. Yield = 100 %, IR (KBr pellet): 3312, 3040, 2921, 2851, 1647, 1621, 1557, 1539, 1506, 1457, 1241, 841, 668, 515cm^−1^; ^1^H NMR (300 MHz, DMSO-d_6_) δ 8.63(t, 2H), 8.35-7.98(m, 18H), 6.07(m, 2H), 5.01(m, 2H), 4.25(t, 2H) 2.96(d, 4H), 1.20(m, 18H).

## Figures and Tables

**Table 1:** Gelation test results of perylenediimide (**1**) at 0.5 equivalent of **2** (1 wt%).

Solvent	State^1^	Solvent	State^1^
Toluene	I	Ethanol	I
H_2_O	I	MC	I
Acetonitrile	I	CHCl_3_	S
Methanol	I	THF	I
Butanol	I	DMSO	G

^1^ G = gel, S = solution, I = insoluble.

## Supplementary Materials

The following are available online at https://www.mdpi.com/2310-2861/6/2/16/s1, Scheme S1: Schematic of synthetic methods for compound **1** and **2**. Figure S1: 1H NMR spectrum of compound **1**. Figure S2: 13C NMR spectrum of compound **1**. Figure S3: IR spectrum of compound **1**. Figure S4: 1H NMR spectrum of compound **2**. Figure S5: IR spectrum of compound **2**. Figure S6: Gelation test results of perylenediimide (1) at 0.5 equivalent of 2 (1wt%); (a) Toluene, (b) H2O, (c) Acetonitrile, (d) Methanol, (e) Butanol, (f) Ethanol, (g) Methylene Chloride, (h) Chloroform, and (i) Tetrahydrofuran. Figure S7: Photographs of (A) sol 1 and (B) sol 2 in DMSO (33.1 mM). Figure S8: The sol-gel transition temperature of co-assembled gel in the presence of (A) 0.5 and (B) 0.8 equiv. of 2. Figure S9: UV-vis spectra of co-assembled supramolecular gel (1 wt%) dependent on the ratio of compound 2 in DMSO. Figure S10: Luninescnece spectra of sol 1 (blue line) and sol 2 (green line) in DMSO (1 wt%); dash line = co-assembled supramolecular gels, solid line = sols. Figure S11: TEM images of co-assembled supramolecular gel dependent on the ratio of compound 2; [2]/[1] = (A) 0.5, and (B) 0.8. Figure S12: SEM image of sol 1 in DMSO (Scale bar = 1 μm). Figure S13: SEM image of sol 2 in DMSO (Scale bar = 1 μm). Figure S14: Rheological properties (G’ black dot; G’’ red dot) of co-assembled supramolecular gel with 0.2 equiv. of 2; frequency sweep tests at 5–1000 Hz and strain 0.1%. Figure S15: Rheological properties (G’ black dot; G’’ red dot) of co-assembled supramolecular gel with 0.5 equiv. of 2; frequency sweep tests at 5-1000 Hz and strain 0.1%. Figure S16: Rheological properties (G’ black dot; G’’ red dot) of co-assembled supramolecular gel with 0.8 equiv. of 2; (A) frequency sweep tests at 5–1000 Hz and strain 0.1%, and (B) continuous step strain test at 0.01% and 1%.
