# Supplementary material for: Pyrene-Based Co-Assembled Supramolecular Gel; Morphology Changes and Macroscale Mechanical Property
Source: Gels. 2020 May 15;6(2):16. doi: 10.3390/gels6020016 (PMC7345282; doi:10.3390/gels6020016)
Supplement: Supplementary file 1 [file gels-06-00016-s001.pdf]

Supplementary Materials

Pyrene-Based Co-Assembled Supramolecular Gel; Morphology Changes and Macroscale Mechanical Property

Ka Young Kim,[1,3] Mirae Ok ,[1,3] Jaehyeong Kim,[1] Sung Ho Jung,[2] Moo Lyong Seo[1,*] and Jong Hwa Jung[1,*]

[1] Department of Chemistry and Research Institute of Natural Sciences, Gyeongsang National University, Jinju; 52828, Republic of Korea; rk5321@gnu.ac.kr (K.Y.K.), meilai97@naver.com (M.O), rwgjjang7@gnu.ac.kr (J.K)

[2] Department of Liberal Arts, Gyeongnam National University of Science and Technology(GNTECH), Jinju; 52725, Republic of Korea; sungho@gntech.ac.kr

[3] These authors contributed equally in this work.

* Correspondence: mlseo@gnu.ac.kr (M.L.S); jonghwa@gnu.ac.kr; Tel.: +82-55-772-1488 (J.H.J)

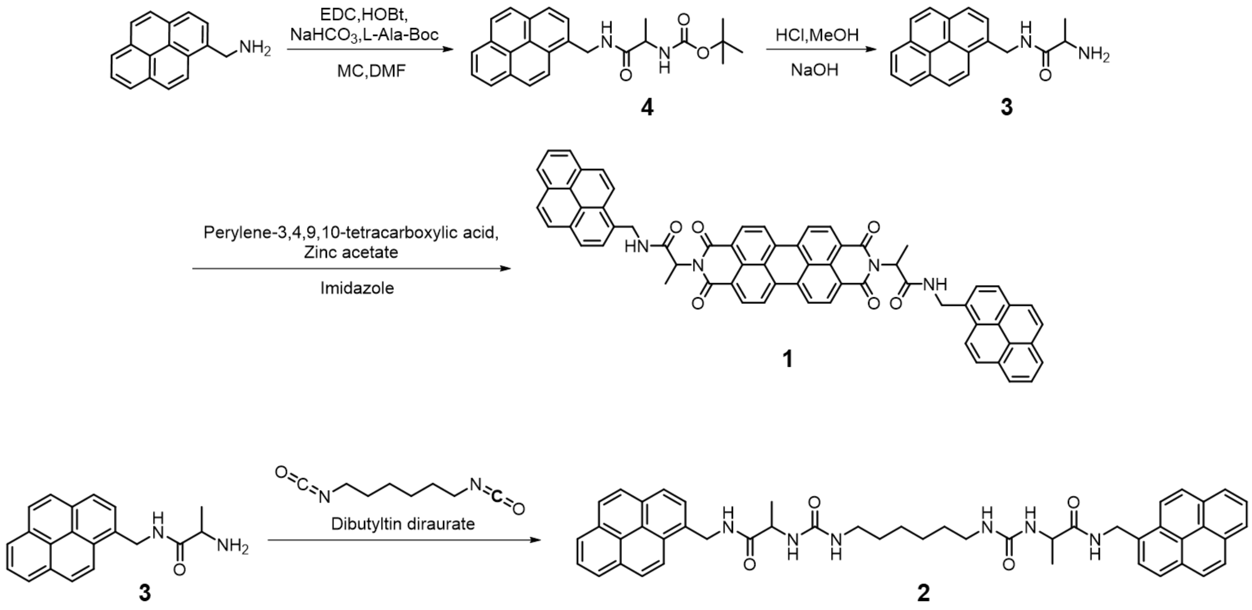

Scheme S1. Schematic of synthetic methods for compound **1** and **2**.

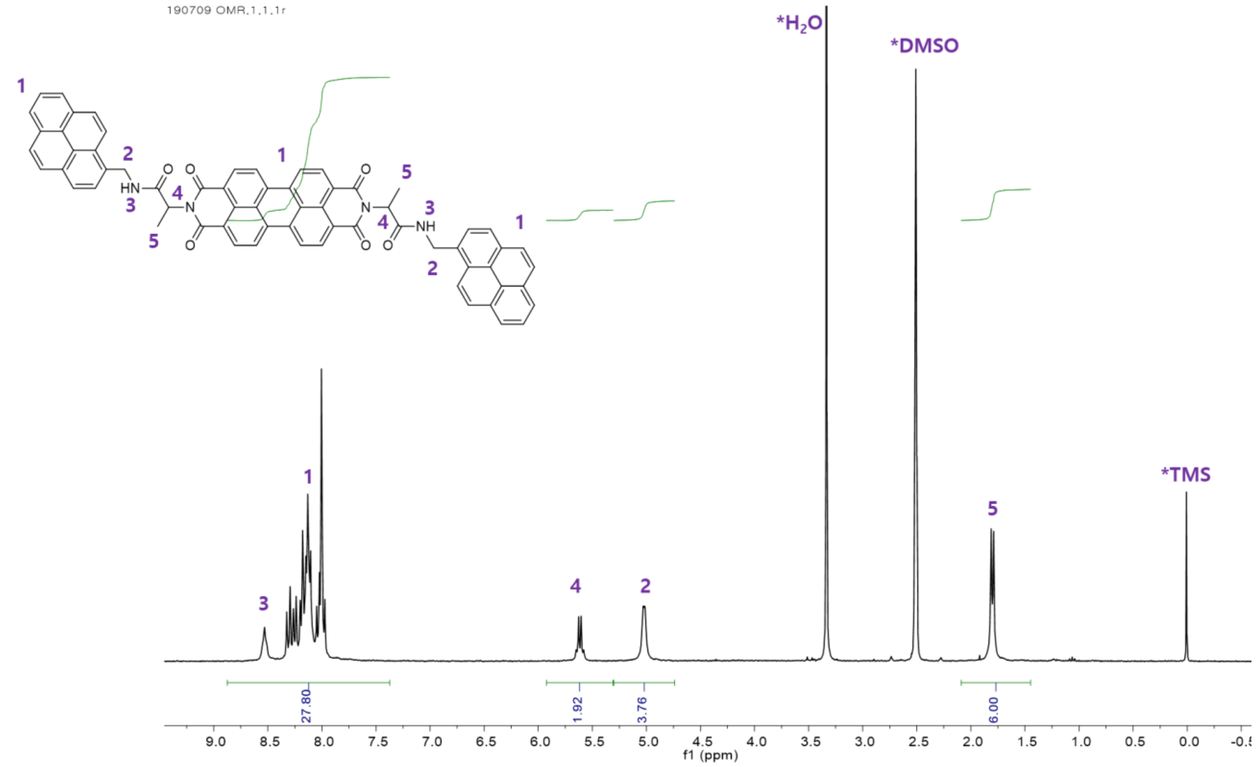

Figure S1. ^{1}H NMR spectrum of compound **1**.

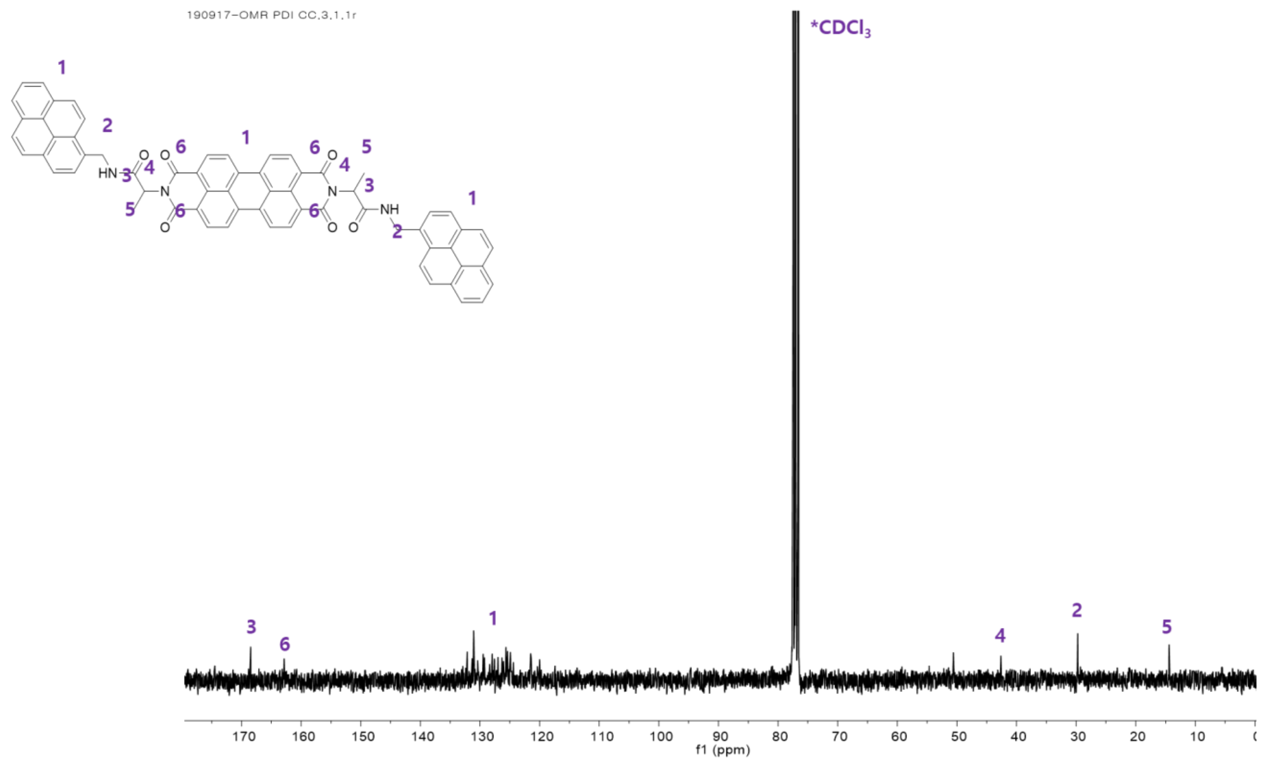

Figure S2. ^{13}C NMR spectrum of compound **1**.

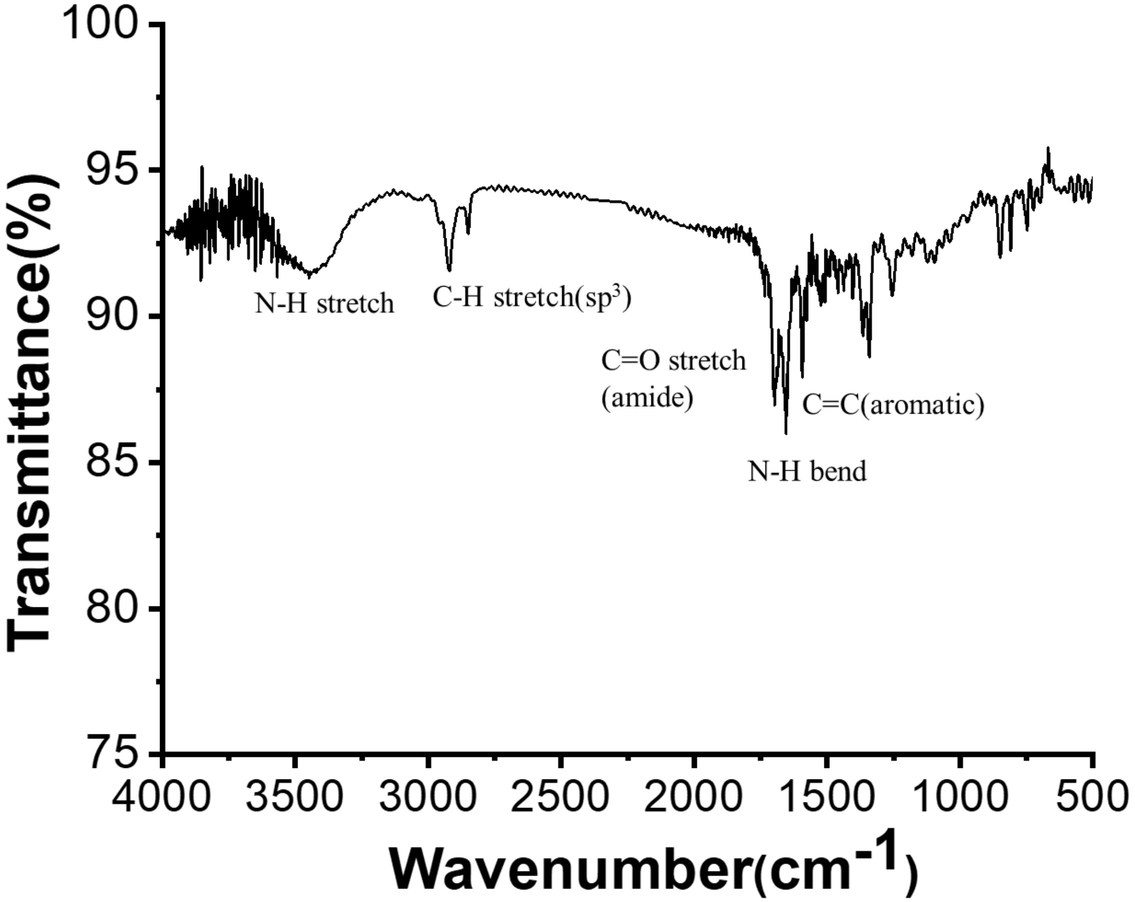

Figure S3. IR spectrum of compound **1**.

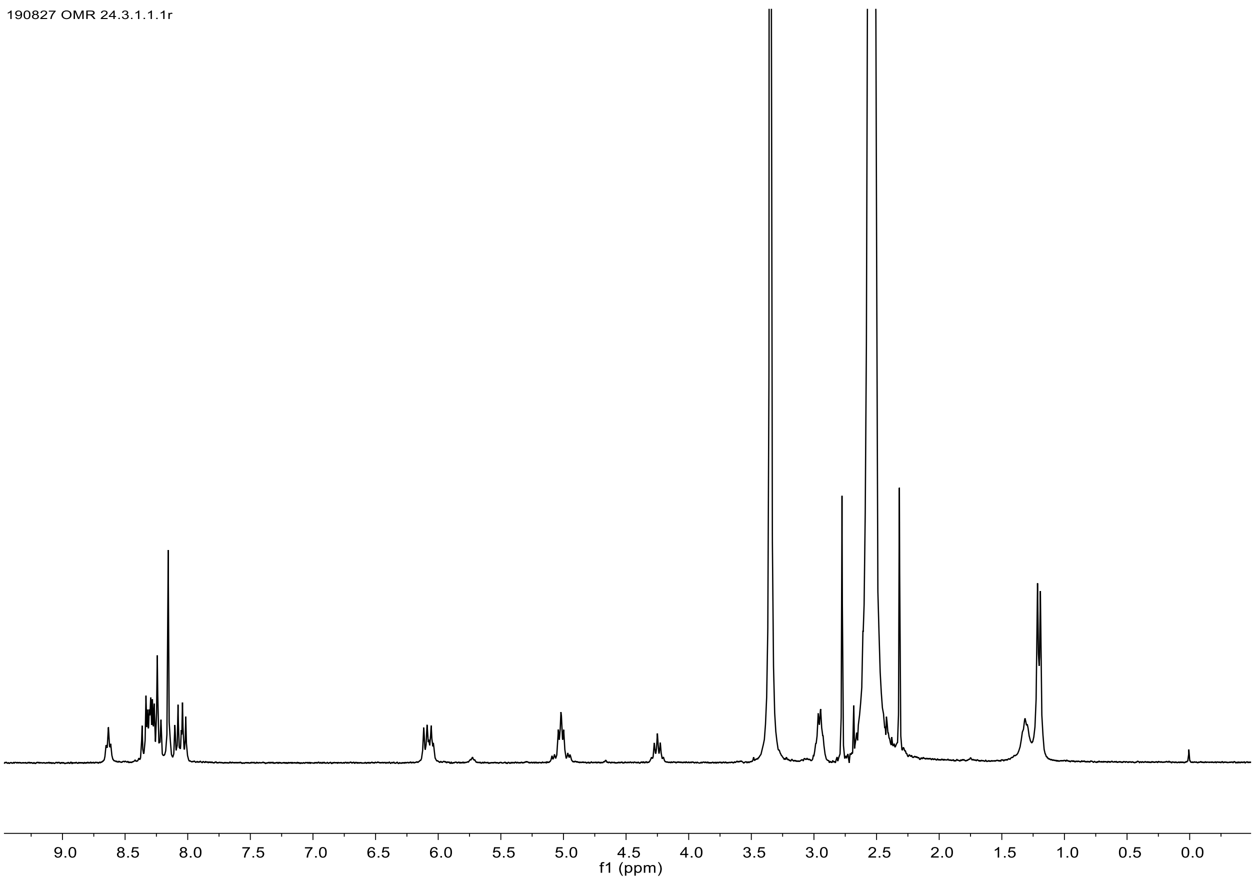

Figure S4. ^{1}H NMR spectrum of compound **2**.

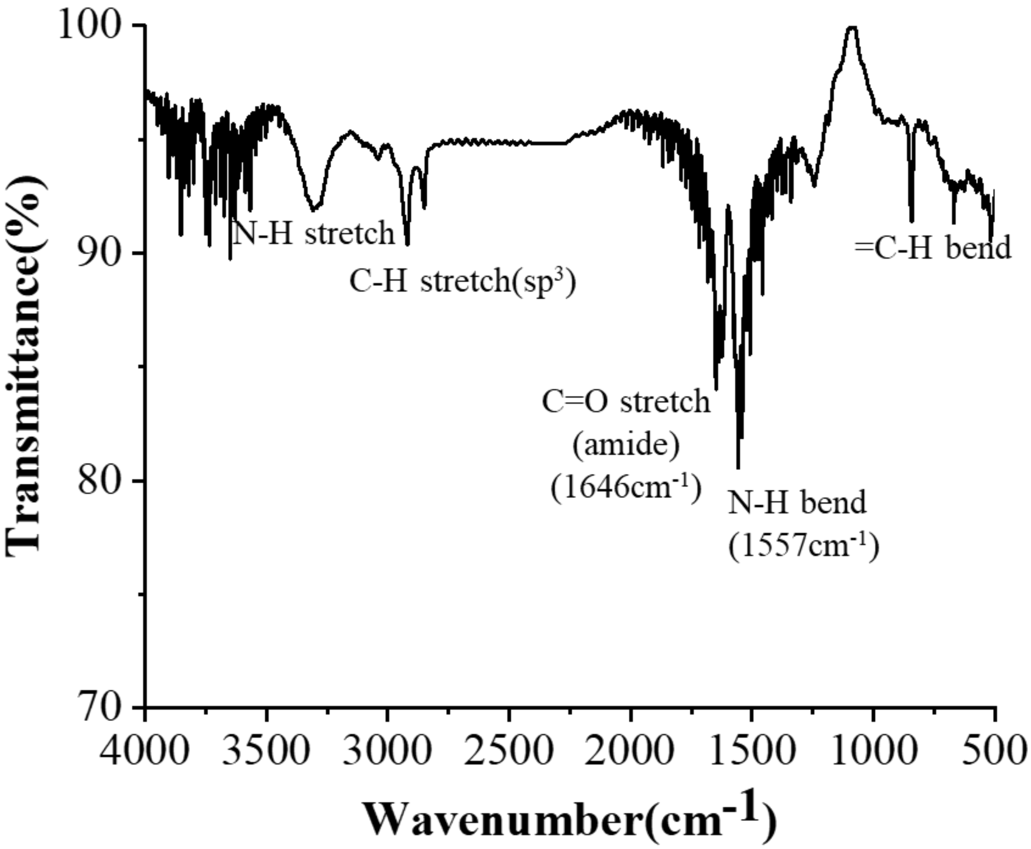

Figure S5. IR spectrum of compound **2**.

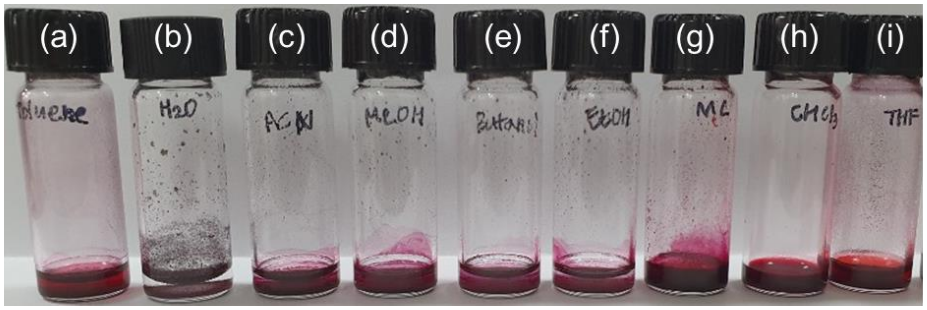

Figure S6. Gelation test results of perylenediimide (**1**) at 0.5 equivalent of **2** (1wt%); (a) Toluene, (b) H_2O, (c) Acetonitrile, (d) Methanol, (e) Butanol, (f) Ethanol, (g) Methylene Chloride, (h) Chloroform, and (i) Tetrahydrofuran.

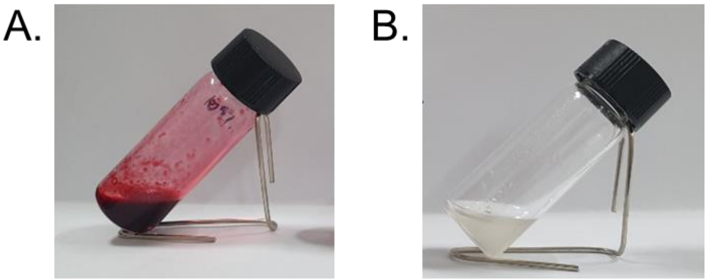

Figure S7. Photographs of (A) sol **1** and (B) sol **2** in DMSO (33.1mM).

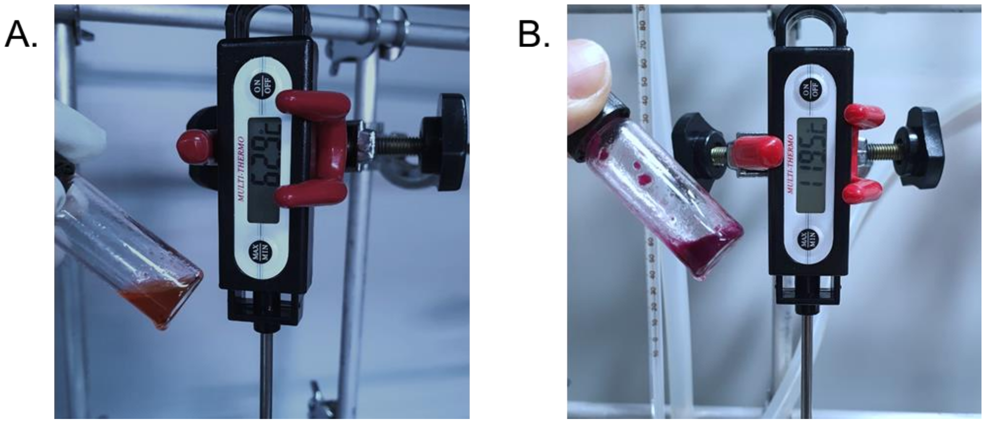

Figure S8. The sol-gel transition temperature of co-assembled gel in the presence of (A) 0.5 and (B) 0.8 equiv. of **2**.

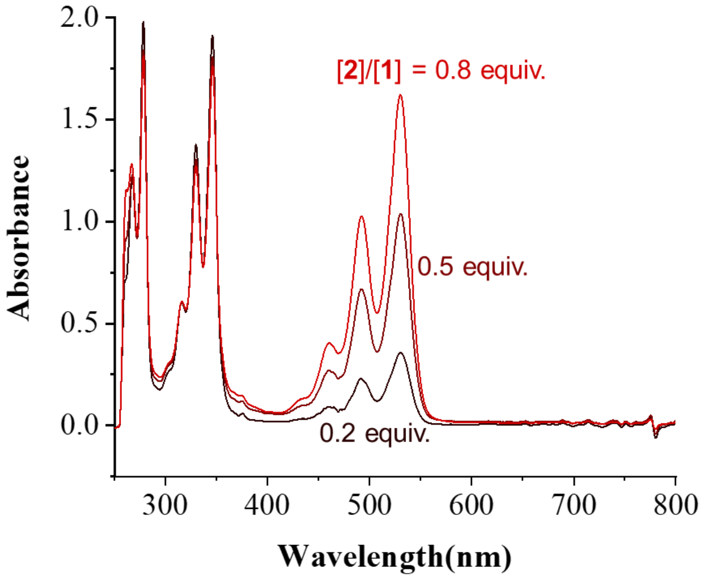

Figure S9. UV-vis spectra of co-assembled supramolecular gel (1 wt%) dependent on the ratio of compound **2** in DMSO.

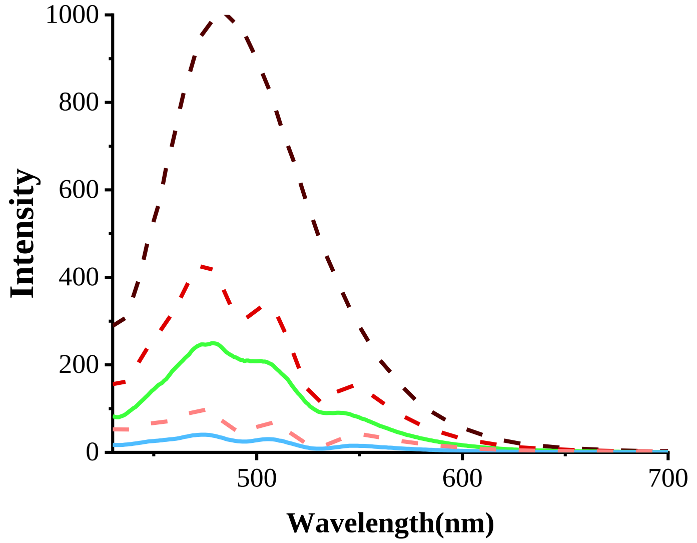

Figure S10. Luninescnece spectra of sol **1** (blue line) and sol **2** (green line) in DMSO (1 wt%); dash line = co-assembled supramolecular gels, solid line = sols.

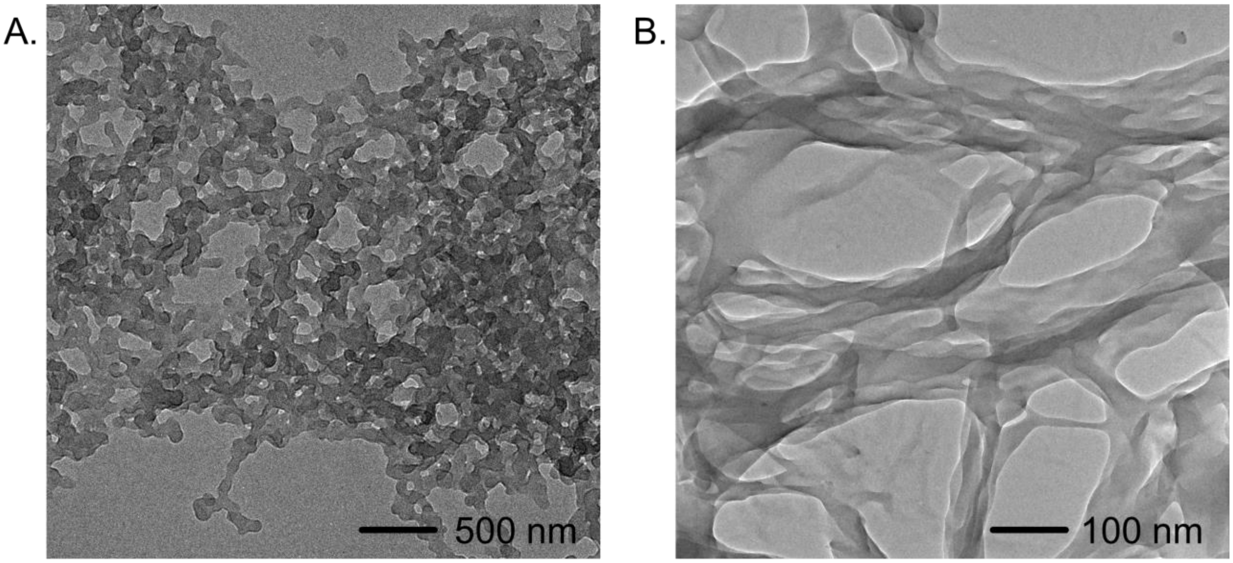

Figure S11. TEM images of co-assembled supramolecular gel dependent on the ratio of compound **2**; [**2**]/[**1**] = (A) 0.5, and (B) 0.8.

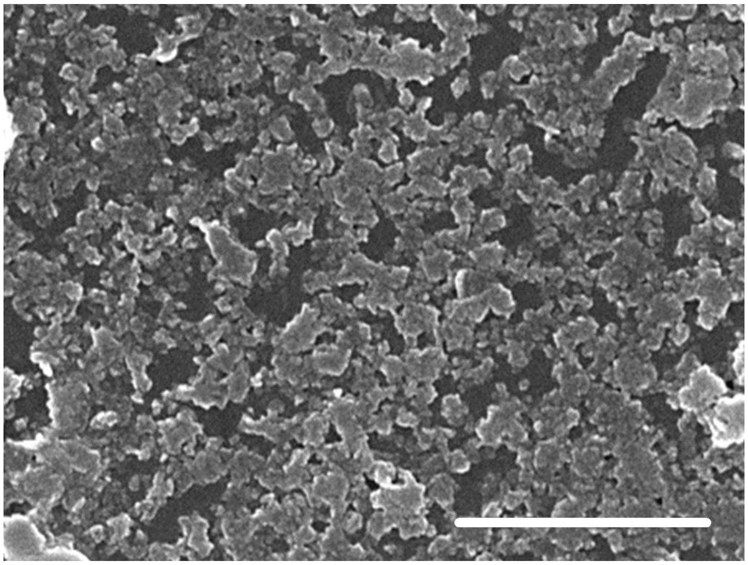

Figure S12. SEM image of sol **1** in DMSO (Scale bar = 1 μm).

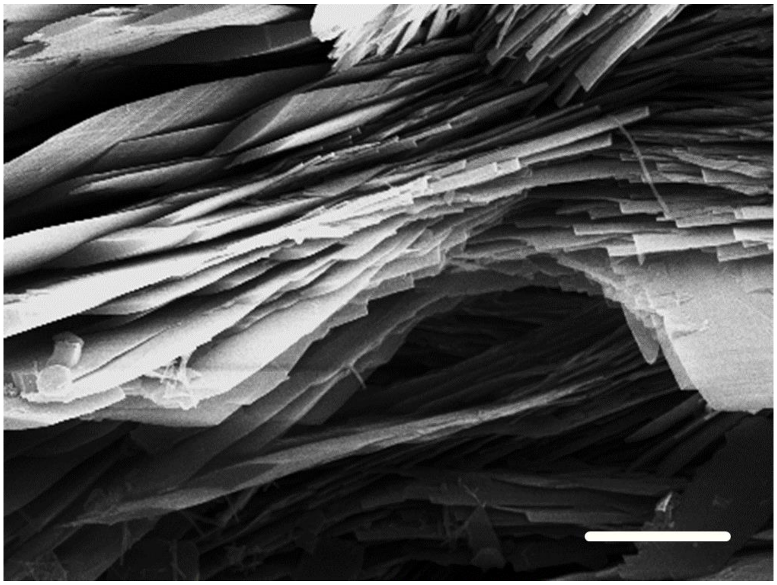

Figure S13. SEM image of sol **2** in DMSO (Scale bar = 1 μm).

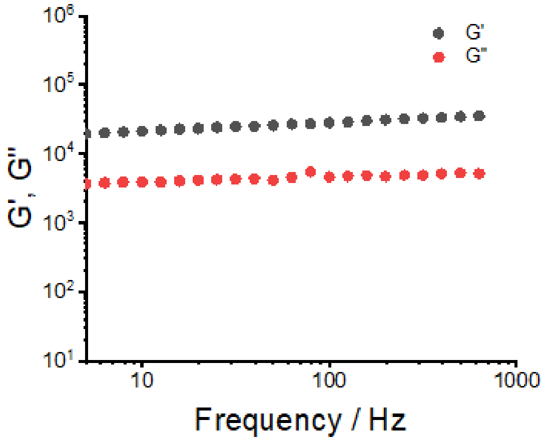

Figure S14. Rheological properties (G’ black dot; G’’ red dot) of co-assembled supramolecular gel with 0.2 equiv. of **2**; frequency sweep tests at 5-1000 Hz and strain 0.1%.

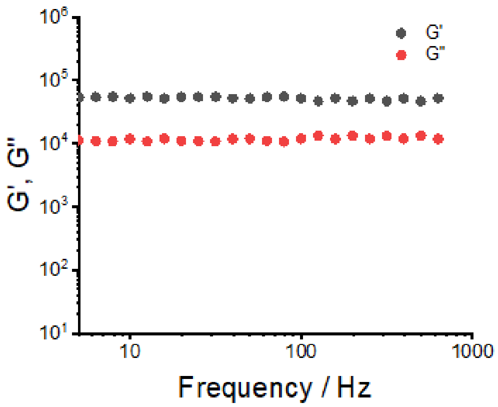

Figure S15. Rheological properties (G’ black dot; G’’ red dot) of co-assembled supramolecular gel with 0.5 equiv. of **2**; frequency sweep tests at 5-1000 Hz and strain 0.1%.

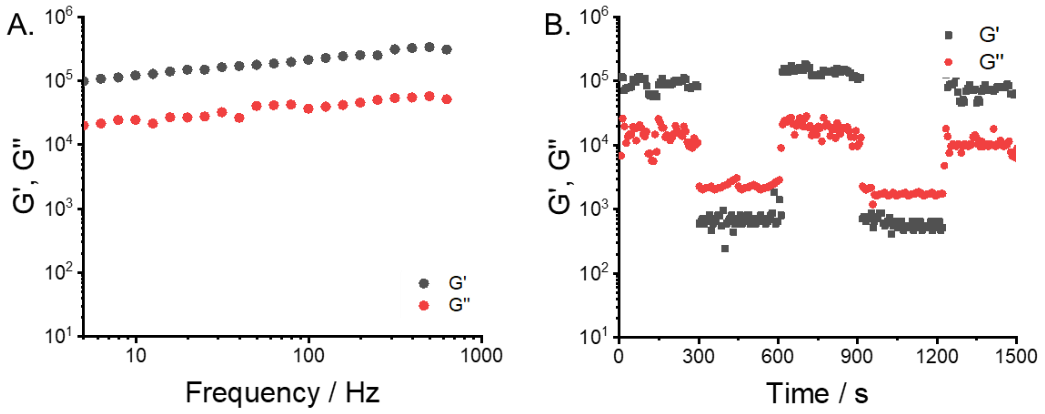

Figure S16. Rheological properties (G’ black dot; G’’ red dot) of co-assembled supramolecular gel with 0.8 equiv. of **2**; (A) frequency sweep tests at 5-1000 Hz and strain 0.1%, and (B) continuous step strain test at 0.01% and 1 %.
